# Molecular diagnosis and preimplantation genetic testing for chromosome 1q21.1 recurrent microduplication

**DOI:** 10.3389/fgene.2025.1522406

**Published:** 2025-03-05

**Authors:** Cuiting Peng, Han Chen, Fan Zhou, Hong Yang, Yutong Li, Yuezhi Keqie, Xu Zhao, He Wang, Ting Hu, Shanling Liu, Jun Ren, Xinlian Chen

**Affiliations:** ^1^ Department of Medical Genetics, West China Second University Hospital, Sichuan University, Chengdu, China; ^2^ Key Laboratory of Birth Defects and Related Diseases of Women and Children, Sichuan University, Ministry of Education, Chengdu, China

**Keywords:** preimplantation genetic testing, microduplication, next-generation sequencing, optical genome mapping, MARSALA

## Abstract

As the development of molecular diagnostic methods, a large number of clinically relevant or disease-related copy number variations (CNVs) could be detected, and the demand for genetic counselling and clinical treatment is also increasing. For patients with pathogenic or likely pathogenic CNVs, preimplantation genetic testing (PGT) could provide a feasible path to prevent the inheritance of the genetic disorder in the offspring. In this study, we included a couple with 1q21.1 recurrent microduplication to conduct molecular diagnosis and PGT clinical application. The optical genome mapping (OGM) successfully verified the orientation and location of the microduplication, which further proved OGM as a promising approach for chromosomal anomalies detection with high resolutions. In PGT application, linkage-analysis-based PGT and high resolution PGT-A were simultaneously conducted for the pedigree and all the embryos. The results were consistent between linkage analysis and high resolution aneuploid analysis in the targeted region. One embryo that was absent of paternal 1q21.1q21.2 duplication was selected for further transplantation. This successful clinical practice in this study shed light for future molecular diagnosis and PGT application in tandem microduplications.

## 1 Introduction

The widespread chromosome copy number variations (CNVs) in the human genome contribute a major source of human genetic diversity, also are associated with rare and complex diseases (Stankiewicz and Lupski, 2010; Cook and Scherer, 2008; Watson et al., 2014). The CNVs size can range from kilobases (kb) to megabases (Mb), which are difficult to be identified by karyotyping analysis (Smeets, 2004). Owing to the development of diagnostic methods such as chromosomal microarray (CMA), whole exome sequencing (WES) and optical genome mapping (OGM), a large number of CNVs, especially those submicroscopic CNVs with ranges less than 5 Mb has been widely identified in recent years (Vissers et al., 2005; Alkan et al., 2011; Kosugi et al., 2019; Schwartz et al., 1993). An increasing number of cases carrying different CNVs has been identified, made it possible to evaluate the correlation of phenotypes with genotypes, and many CNVs were verified to be clinically relevant or disease-related (Wang et al., 2017). And spontaneously, interpretation of clinical significance and genetic counselling for CNVs has become more and more complicated.

Recurrent rearrangements of chromosome 1q21.1 was one subset of CNVs that has been reported in association with variant clinical phenotypes (Mefford et al., 2008). The chromosome 1q21.1 locus, with multiple low-copy repeats, is susceptible to recurrent deletions and duplications. Carriers of the 1q21.1 recurrent microduplications (with a minimal duplication size of ∼1.35 Mb) present variable phenotypes ranging from normal clinical features to developmental delay (DD), autism spectrum disorders (ASDs), seizures and congenital anomalies (Kaminsky et al., 2011; Qiao et al., 2017; Huang et al., 2023). The diverse phenotypes and incomplete penetrance, made genetic counselling for 1q21.1 microduplication challengeable.

Once a pathogenic CNV is recognized in a couple, prenatal diagnosis or preimplantation genetic testing (PGT) can be utilized to prevent the transmission of the pathogenic CNV (Geraedts and Sermon, 2016; Sabria-Back et al., 2022). Recently, PGT strategy based on SNP haplotyping has been applied in couples with small pathogenic CNVs to identify unaffected embryos, since the conventional PGT-A platform is not fully available because of the limited resolution (Xie et al., 2022).

In this study, we performed PGT for a couple with an approximately 1.69 Mb of duplication in chromosome 1q21.1q21.2 region. We first validated that the duplication is arranged in tandem arrays by OGM, then we performed SNP-based PGT and high-resolution PGT-A for the embryos. A total of 5 embryos were obtained and 2 of them were absence of the inherited duplication, of which 1 embryo was selected for transplantation after further PGT-A analysis. Our results validated the significant advantages of OGM in detecting small CNVs, especially to identify the locations of microduplications. Also, the results verified the value of linkage-analysis -based PGT-M application in tandem microduplications.

## 2 Methods

### 2.1 Molecular diagnosis by optical genome mapping

Peripheral blood sample was collected and ultrahigh molecular weight DNA was extracted from the man with 1q21.1q21.2 duplication via the SP Blood and Cell Culture DNA Isolation Kit (Bionano Genomics, San Diego, CA, United States). Subsequently, genomic DNA sample were labelled with DLS DNA Labeling Kit (Bionano Genomics), loaded on a Saphyr chip and imaged on the Saphyr instrument following the manufactory’s directions. Data collection and analysis were performed with Bionano Solve software v.3.5 (Bionano Genomics), Bionano Access software (version1.7.1) (Bionano Genomics), as we described previously (Ren et al., 2023). The reference for analysis is hg19_DLE1_0 kb_0labels.cmap and map rate for this case is around 93.7%. For CNVs, the data calls were output and annotated with confidence score (set at 0.99) and feature CNV overlap precision was over 500 kb (Dremsek et al., 2021).

### 2.2 *In vitro* fertilization, trophectoderm biopsy and whole genome amplification (WGA)

Ovarian stimulation, *in vitro* fertilization and later embryo transfer processes after genetic testing were conducted in the reproductive medical center according to the standard protocol (Schoolcraft et al., 2011; McArthur et al., 2005). Trophectoderm (TE) biopsy were conducted for a total of 5 embryos. Two-steps of whole genome amplification method of multiple annealing and looping-based amplification cycles (MALBAC) (Yikon Genomics) was applied in WGA for each embryo following the standard protocol (Zong et al., 2012). Whole genome products were then purified using DNA Clean-up Kit (CWBIO).

### 2.3 SNP haplotyping analysis based on MARSALA

Genomic DNA was extracted from both peripheral blood samples of this couple and amniotic fluid cells from the affected offspring according to the manufacturer’s instructions (QIAGEN, QIAamp DNA Micro Kit). Then, the mutated allele revealed by sequencing with aneuploidy and linkage analyses (MARSALA) were conducted for all the samples (Yan et al., 2015). Around 5 Mb of valid reads were collected for each sample by sequencing on the MGI 200 or T7 platform. An average sequencing depth of 2-3× were obtained, which is sufficient for measuring call-targeted SNPs (Xiong et al., 2019). Raw reads were filtered by fastp and aligned to human reference genome (hg19) (BWA). Bam files were then exported after marking duplicates (markdup) by samblaster program. Variant calling and SNP annotation were conducted using bcftools. SNPs with a depth of less than 4× or with low quality were filtered out. The heterozygous SNPs with frequencies outside the range of 0.2–0.8 and homozygous within the range of 0.1–0.9 were filtered out. Haplotyping analysis was then conducted using informative SNPs which were heterozygous in the male (the microduplication carrier) and homozygous in the female (the normal spouse). Particularly in this case, the same and homozygous SNPs inside the microduplication region for the female and the affected offspring, could also be selected for linkage analysis. MARSALA procedure and haplotyping analysis were then conducted for embryos.

### 2.4 Sanger sequencing validation

To validate the detection efficiency of MARSALA, a total of 5 informative SNPs from both upstream and downstream of microduplication region were selected to perform Sanger sequencing validation. Five pairs of specific primers were designed to amplify the segments containing the SNPs sites. PCR amplifications were performed as before (Peng et al., 2023). Subsequent Sanger sequencing was performed and data was analyzed using ChromasPro software.

### 2.5 CNV analysis and direct mutation detection by MARSALA

The NGS data obtained based on MARSALA platform for each embryo was sufficient for whole genome CNV analysis, even for precisely detection of 1.69 Mb duplication in this case (Yan et al., 2015). Valid reads for each sample were around 5 Mb. The raw reads were filtered by fastp (https://github.com/OpenGene/fastp) and aligned to reference sequence using BWA software. For >4 Mb CNVs analysis, the reads were counted with the bin size of 1 Mb. For analysis of small CNVs (around 1 Mb) in target region, 400 kb of bin size was divided. Circular binary segmentation algorithm (CBS) and R-language were used for copy number variations reporting and visualization for the whole genome based on each bin.

## 3 Results

### 3.1 Patient and genetic background

**FIGURE 1:**
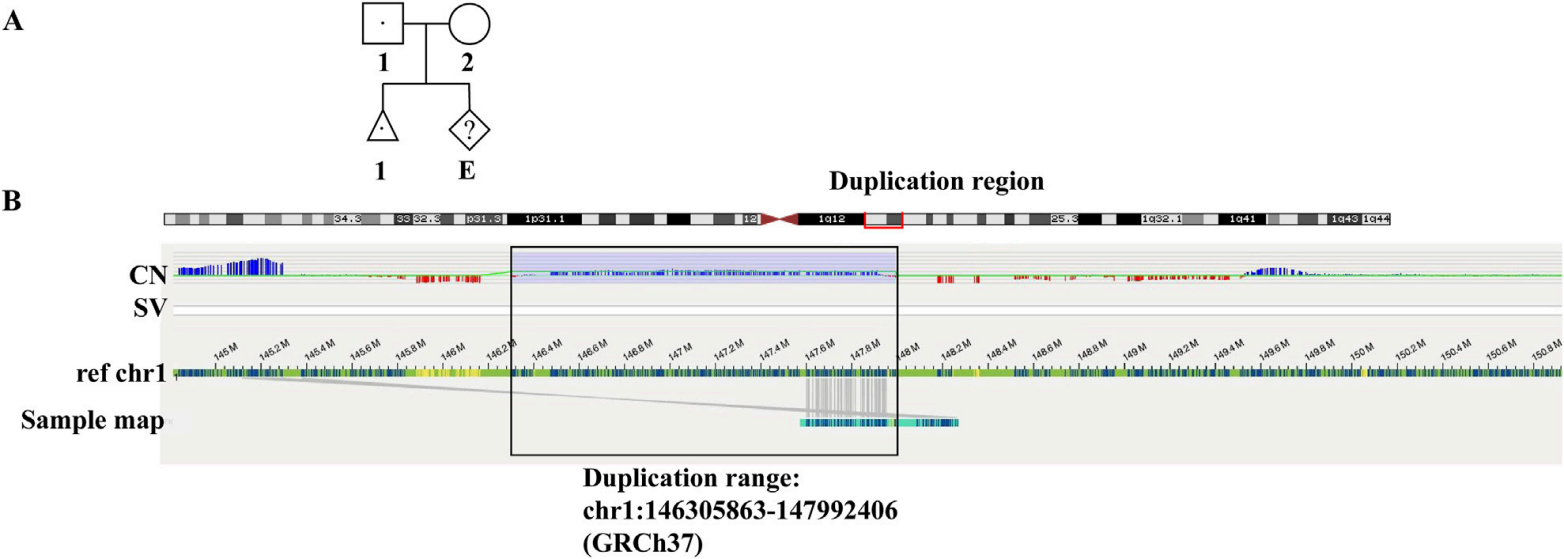
Pedigree of family **(A)** and the OGM results **(B)**. **(A)** In the pedigree, the affected fetus inherited the paternal mutated allele of microduplication. **(B)** The red box on the top showed the region of duplication in chromosome 1. The black box showed the duplication range of chr1:146305863-147992406 (GRCh37) based on CNV plot (CN) and sample genome map against reference chromosome 1.

A couple with advanced maternal and paternal ages (both >35 years old) who have experienced recurrent miscarriage (RM) was included in this study (Figure 1A). The phenotypes and karyotypes were both normal for the couple. Chromosomal microarray analysis for amniotic fluid cells from the most recent pregnancy detected a 1,690 kb of microduplication in 1q21.121.2 region, which is paternal inherited (data not shown here). After sufficient genetic counselling and fully informed consent, this couple hope to avoid the inheritance of this microduplication by the help of PGT, in spite that no definite phenotypes were described in the male carrier.

### 3.2 Molecular diagnosis of 1q21.1 tandem microduplication by optical genome mapping

To successfully apply SNP haplotyping-based PGT in this case, OGM analysis was applied to further accurately locate the duplication segment. The results showed a microduplication of around 1.69 Mb in chr1 (q21.1-q21.2) as shown in Figure 1B. The red box on the top showed the region of microduplication in chromosome 1. CNV plot and sample genome map against reference showed the exact duplication range of chr1:146305863-147992406 (GRCh37) (black box). The genome-wide circos plot exhibited the whole chromosomes and the CNV profile represented by the blue line in the black box highlighted the microduplication in chromosome 1 (Supplementary Figure S1). No evidence showed the microduplication inserted in other chromosome by OGM. The results verified the duplication was a tandem repeat nearing the original location in 1q21.1.

### 3.3 SNP haplotyping and sanger sequencing validation

**FIGURE 2:**
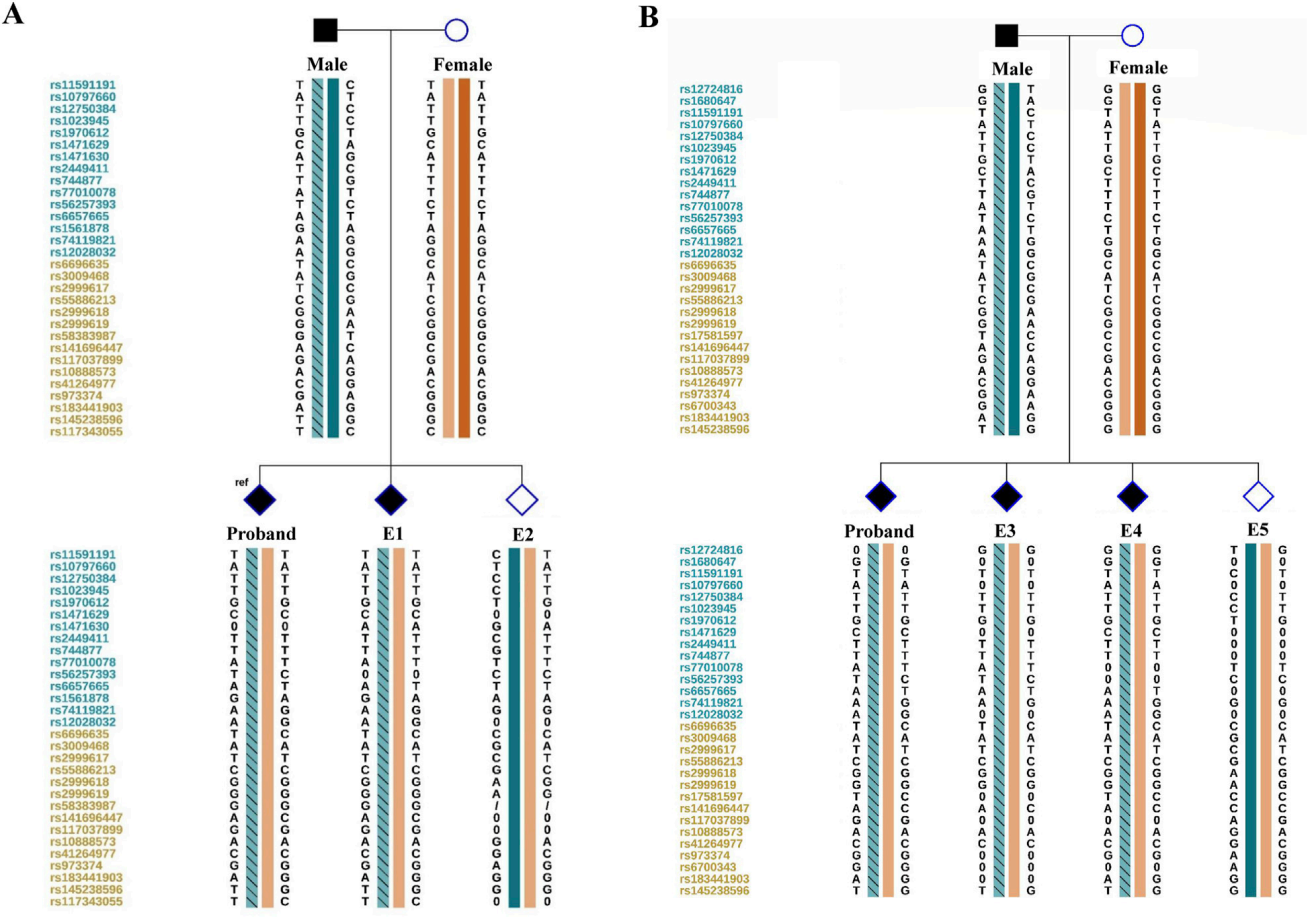
Schematic representative of haplotype linkage analysis for the pedigree and embryos from two cycles **(A, B)**. Male and female haplotypes were highlighted in different colors. The haplotype of light blue frame with slashes refer to the high-risk haplotype of the male deduced by linkage analysis. The reference SNP cluster ID numbers were listed on the left side. The ID numbers highlighted in dark blue and orange refer to the upstream and downstream informative SNPs respectively.

Once the molecular diagnosis of 1q21.1 tandem microduplication was verified, the PGT strategy based on SNP haplotyping would be applicable. In this study, we first conducted next-generation sequencing for the couple and the affected offspring based on MARSALA platform. Haplotyping analysis was then conducted using informative SNPs within the 2-million base pair (Mbp) region flanking the target microduplication region (chr1:146305863-147992406). The representative informative SNPs used in linkage analysis were listed in Table 1. Paternal hap 1 represented the high-risk haplotype linked with the 1q21.1 microduplication, and hap 2 represented the low-risk haplotype on the contrary. Notably, in microduplication region, these heterozygous SNPs in the male while same and homozygous in the female and the affected offspring, could also be selected for linkage analysis as showed in Table 1 (marked with grey shading). For these SNPs, affected offsprings should be homozygous same as the proband, while unaffected offsprings should be heterozygous. In clinical cycle, embryos were then conducted linkage analysis after WGA and sequencing. A total of 5 embryos was obtained after two IVF cycles and two of them (E2 and E5) were verified that not inherited the paternal high-risk haplotype based on SNP linkage analysis (as shown in Figure 2; Table 1). Five SNPs sites were selected to further conduct Sanger sequencing on all the samples to validate the accuracy of NGS (data not shown here).

**TABLE 1:** Representative informative SNPs used in linkage analysis.

RSID	POS	Distance	Male	Paternal hap 1	Paternal hap 2	Female	Proband	E1	E2	E3	E4	E5
rs1747899	144,939,820	−1366043	C/G	C	G	C/C	C/C	C/C	C/G	C/C	C/C	C/G
rs117176,741	145,555,016	−750847	A/T	T	A	A/A	A/T	A/T	A/A	A/T	A/T	A/A
rs11804687	145,569,027	−736836	A/C	C	A	C/C	C/C	C/C	A/C	C/C	C/C	A/C
rs10910830	145,587,540	−718323	C/G	G	C	C/C	C/G	C/G	C/C	C/G	C/G	C/C
rs3754342	145,595,542	−710321	G/C	C	G	G/G	G/C	G/C	G/G	G/C	G/C	G/G
rs11591191	145,675,931	−629932	T/C	T	C	T/T	T/T	T/T	T/C	T/T	T/T	T/C
rs12750384	145,681,484	−624379	T/C	T	C	T/T	T/T	T/T	T/C	T/T	T/T	T/C
rs1023945	145,703,115	−602748	T/C	T	C	T/T	T/T	T/T	T/C	T/T	T/T	T/C
rs1970612	145,703,590	−602273	G/T	G	T	G/G	G/G	G/G	G/T	G/G	G/G	G/T
rs74696952	146,791,849		T/C			T/T	T/T	T/T	T/C	T/T	T/T	T/C
rs117246310	146,793,088		T/G			T/T	T/T	T/T	./	T/T	T/T	T/G
rs2353991	146,933,543		T/A			A/A	A/A	A/A	T/A	A/A	A/A	T/A
rs74609846	147,045,875		C/G			C/C	C/C	C/C	C/G	C/C	C/C	C/G
rs183547578	147,186,471		C/T			C/C	C/C	C/C	./	C/C	C/C	C/T
rs3009468	147,825,454		A/G			A/A	A/A	A/A	A/G	A/A	A/A	A/G
rs2999617	147,825,662		T/C			T/T	T/T	T/T	T/C	T/T	T/T	T/C
rs55886213	147,825,732		C/G			C/C	C/C	C/C	C/G	C/C	C/C	C/G
rs2999618	147,825,763		G/A			G/G	G/G	G/G	G/A	G/G	G/G	G/A
rs2999619	147,825,765		G/A			G/G	G/G	G/G	G/A	G/G	G/G	G/A
**rs17581,597**	**149,860,372**	**1,867,966**	C/T	T	C	C/C	C/T	C/T	C/C	C/T	C/T	C/C
**rs141696447**	**149,903,396**	**1,910,990**	C/A	A	C	C/C	C/A	C/A	C/C	C/A	C/A	C/C
**rs117037899**	**149,934,524**	**1,942,118**	G/A	G	A	G/G	G/G	G/G	G/A	G/G	G/G	G/A

RSID: reference SNP cluster ID, the ID numbers highlighted in bold refer to the downstream of duplication region;

POS: genomic location;

Distance: distance flanking the duplication region (chr1: 146305863-147992406 by OGM)

Reference sequence: GRCh37/hg19 reference genome;

Paternal hap1 and hap 2 represent high-risk and low-risk haplotype of the male respectively.

Areas marked with grey shading showed the haplotype analysis in the duplication region.

### 3.4 CNV analysis

**FIGURE 3:**
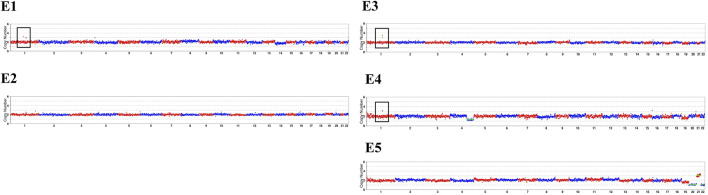
Copy number variations results of the pedigree and embryos. The sketch maps are generated after compared with the hg19 reference genome based on the MARSALA data. The black box highlights the duplication in chromosome 1 of E1, E3 and E4. The red box refers to different chromosomal abnormalities in E4 and E5. The detailed CNVs results were presented in Table 2.

**FIGURE 4:**
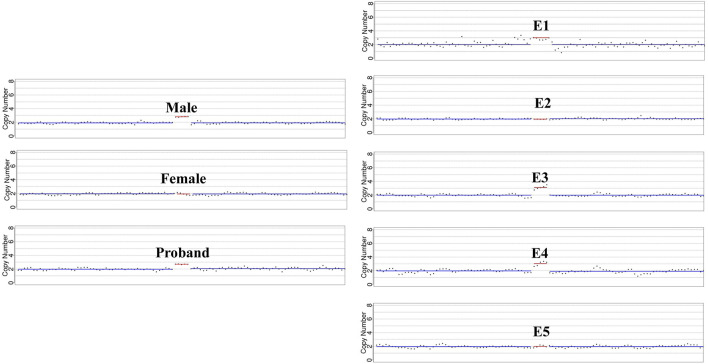
Detailed microduplication detection in targeted region. The sketch maps highlight the copy number variations in chromosome 1q21.1q21.1 based on direct microduplication detection. Male, the affected proband, embryo E1, E3 and E4 showed a copy number of 3 in the targeted region.

In our conventional PGT-A procedure, only deletions or duplications greater than 4 Mb will be reported when reads are aligned. In this study, CNVs greater than 1 Mb will be presented after CNV analysis of the specific duplication case. The CNVs results were summarized in Table 2, and sketch maps were displayed in Figure 3. As the results shown in Table 2; Figure 3, the embryo E4 showed a deletion of around 53 Mb in region of 4q28.3q35.2 other than the microduplication of 1q21.1q21.2. Embryo E5 showed chromosomal number abnormalities of chromosome 20, 21 and 22, though without microduplication in 1q21.1q21.1. On the contrary, embryo E1 and E3 inherited the paternal microduplication, although there are no other chromosomal abnormalities. Thus, only embryo E2 was normal karyotype and absent of 1q21.1q21.1 microduplication. Direct duplication detection also showed that E1, E3 and E4 exhibited a copy number variations in the target region (Figure 4). In summary, based on SNP haplotyping and CNV analysis, embryo E2 could be selected for subsequent transplantation after the PGT analysis.

**TABLE 2:** Detection results summary of the five biopsied blastocysts.

Biopsied blastocysts	Grade of blastocysts	CNV analysis and direct duplication detection	SNP Linkage analysis	SNP Sanger sequencing validation
E1	5BC	dup (1)(q21.1q21.2)(∼1.20 Mb)	Affected	accordance
E2	4BB	Normal	Unaffected	accordance
E3	4BB	dup (1)(q21.1q21.2)(∼1.20 Mb)	Affected	accordance
E4	4BC	dup (1)(q21.1q21.2)(∼1.20 Mb) del(4)(q28.3q35.2)(∼53.00 Mb)	Affected	accordance
E5	4BC	−20,+21,-22	Unaffected	accordance

CNV, copy number variation detected by MARSALA.

## 4 Discussion

Traditionally, NGS-based PGT-A was applied for genome-wide aneuploidy analysis with resolution of >5 Mb (Group EP-SP-AW et al., 2020). And the PGT-M strategy based on linkage analysis was widely used in monogenic disorders to distinguish high-risk and low-risk haplotype of a prospective parent (Group et al., 2020). For small CNVs, especially those <1 Mb, conventional PGT-A strategy is not available. Inspired by PGT-M strategy, linkage analysis based on upstream and downstream informative SNPs of the target regions could also be used for PGT analysis in couples with small CNVs, once a CNV was recognized as pathogenic or likely pathogenic. For PGT in microdeletions, linkage analysis could be conducted based on informative SNPs flanking or within the deletion regions detected by NGS or SNP array, together with high resolution PGT-A for aneuploidy analysis (Hu et al., 2024).

The situation is more complicated for PGT in microduplications, since SNP-based linkage analysis could only be available when the duplications are precisely located in chromosome. The molecular diagnosis for microduplication, especially to decipher the orientation and location of the duplicated segments, is usually challengeable using karyotype, CMA or NGS platform due to their limited resolution, short read length or other technical limitations. The long-read sequencing technologies could generate long and contiguous reads to cover longer genomic regions, which is potential for direct haplotype phasing or precisely breakpoint detection (Peng et al., 2023; Zhang et al., 2019; Thibodeau et al., 2020). Long reads are superior to short reads regarding detection break points of microdeletions/duplications to the kilobase level. Yet, the detection accuracy and efficiency will be significantly decreased for larger microdeletions/duplications. Another new method named C-MoKa (chromosome conformation-based karyotyping) is reported as a promising method for chromosomal abnormalities detection, especially for complex and cryptic SVs (Bao et al., 2025). Optical genome mapping (OGM) based on whole-genome imaging and assembly, has recently been proved as a valuable approach for chromosomal anomalies detection with high resolutions. Ultra-high-molecular-weight DNA was labeled at specific sites with an average distance about 3.6 kb and genome assembly was performed, made it possible for OGM to detect small SVs and unbalanced aberrations at sizes ranging from a few kilobases to several megabases (Dremsek et al., 2021). Thus, OGM could be a promising diagnostic approach for chromosome microduplications, including detection of the duplication size, orientation and location.

In this study, a workflow of PGT for microduplication was depicted including molecular diagnosis by OGM, linkage analysis and PGT-A by NGS. At first, OGM analysis was conducted for the male 1q21.121.2 microduplication carrier (detected by CMA) before the couple can be included in linkage-analysis-based PGT strategy. Combined with CNV and SV calling by OGM, a 1.69 Mb of tandem microduplication was found in 1q21.121.2 region. The results further validated OGM as an efficient method for small CNVs detection. Then, in consideration of the duplication size, a high genomic resolution of next-generation sequencing was conducted for all the samples to fully detect SNPs and small chromosomal anomalies. This so-called MARSALA method with adequate and flexible resolution, provides an economic and timesaving approach in PGT applications for small CNVs. In this study, informative SNPs were selected for linkage analysis and high resolution of PGT-A was simultaneously conducted for all the embryos. Furthermore, to validate the detection efficiency of MARSALA, a total of 5 informative SNPs were selected to perform Sanger sequencing for validation. The PGT results in this case verified the accordance of SNP-based linkage analysis with high-resolution PGT-A for small CNVs.

It is worth mentioning that for this couple with advanced ages, few embryos were obtained from each IVF cycle, made the chances of obtaining normal embryos less likely. Only embryo E2 was normal karyotype and absent of paternal 1q21.1q21.1 duplication, and ready for transplantation. Embryo E1 and E3 both inherited the paternal duplication while without other chromosomal abnormalities. After genetic counselling, this couple decided to freeze embryo E1 and E3 for later consideration since they are with no willing for further IVF cycle due to advanced ages. Notably, in PGT application, genetic counselling is essential for patients to be informed of the probability for multiple IVF cycles or no suitable embryos to transfer, especially for patients with advanced ages (Murphy et al., 2019; Parikh et al., 2023). On the other hand, the heterogeneities in phenotypes made small CNVs as a clinical dilemma including diagnosis, variation interpretation and genetic counselling. The development of PGT provides a feasible path for patients with genetic deficiency to prevent the recurrence of the disorder in the next-generation. And our strategy of molecular diagnosis and PGT in this study, provided a successful clinical practice and shed light for future applications of PGT in microduplications.

## Data Availability

The raw data supporting the conclusions of this article will be made available by the authors, without undue reservation.

## Supplementary material

SUPPLEMENTARY FIGURE S1 Exported circos plot of OGM analysis. The genome-wide circos plot showing the whole chromosomes after OGM analysis. The blue line in the black box showed the duplication in chromosome 1.
