# Supplementary figures and images for: Molecular diagnosis and preimplantation genetic testing for chromosome 1q21.1 recurrent microduplication
Source: Front Genet. 2025 Mar 5;16:1522406. doi: 10.3389/fgene.2025.1522406 (PMC11919917; doi:10.3389/fgene.2025.1522406)

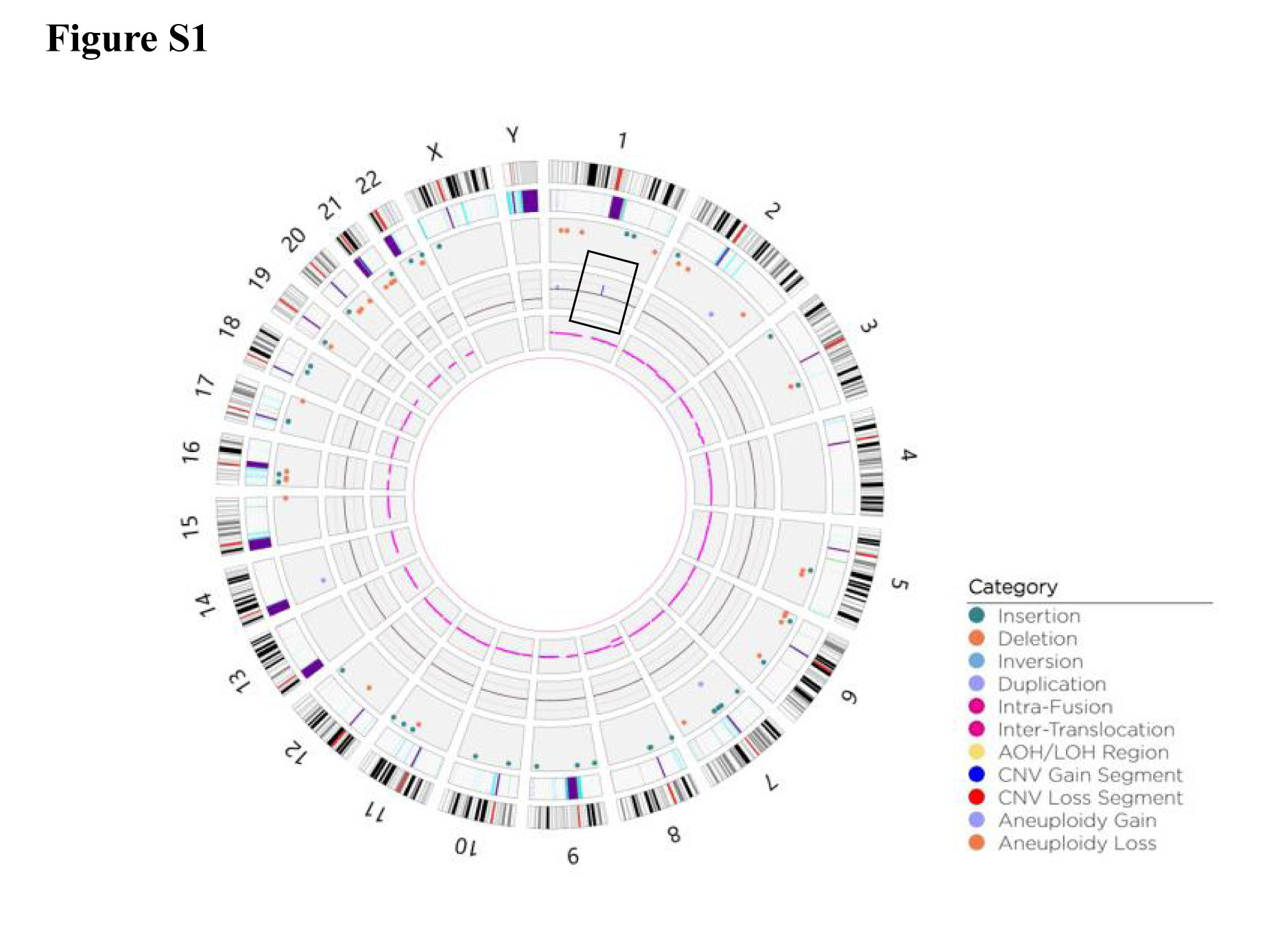

Supplement: Supplementary file 1 [file Image1.tif]
